# The interplay between HPV, other Sexually Transmissible Infections and genital microbiome on cervical microenvironment (MicroCervixHPV study)

**DOI:** 10.3389/fcimb.2023.1251913

**Published:** 2024-03-12

**Authors:** J. Guilherme Gonçalves-Nobre, Andreia Matos, Mariana Carreira, Ana Carolina Santos, Luisa Carvalho Veiga, Catarina Ginete, Miguel Brito, Marina Pires, Hermínia Pereira, Carlos Cardoso, Manuel Bicho, Maria Clara Bicho

**Affiliations:** ^1^ Institute of Environmental Health (ISAMB), Faculdade de Medicina, Universidade de Lisboa, Lisboa, Portugal; ^2^ Institute of Preventive Medicine and Public Health (IMPSP), Faculdade de Medicina, Universidade de Lisboa, Lisboa, Portugal; ^3^ PTSurg – Portuguese Surgical Research Collaborative, Lisbon, Portugal; ^4^ Instituto de Investigação Científica Bento da Rocha Cabral, Lisboa, Portugal; ^5^ Tumour & Microenvironment Interactions Group i3S- Instituto de Investigação e Inovação em Saúde, Universidade do Porto, Porto, Portugal; ^6^ ICBAS – Instituto de Ciências Biomédicas Abel Salazar, Universidade de Porto, Porto, Portugal; ^7^ Amedes MVZ Müenchen GmbH fier Gynaekologie und Pathologie, Munich, Germany; ^8^ Health and Technology Research Center, Escola superior de Tecnologia da Saúde de Lisboa, Instituto Politécnico de Lisboa, Lisbon, Portugal; ^9^ Joaquim Chaves Saúde, Laboratório de Análises Clínicas, Carnaxide, Portugal

**Keywords:** cervicovaginal microbiota, HPV, cytokines, blood count ratios, cervical neoplasia, cervical intra-epithelial neoplasia

## Abstract

**Background:**

The importance of Cervicovaginal Microbiota in protecting against infections (such as HPV) is already well established, namely through Lactobacillus spp., as well as the mechanism through which HPV leads to Cervical Neoplasia. However, it is not possible to classify HPV as a complete carcinogen. Thus, the importance of exploring Cervicovaginal dysbiosis with the intention of deciphering this interaction with HPV, takes on greater relevance. The main objectives of this study were: 1) Comparison of the MCV composition of women with or without HPV and women with ASCUS or LSIL; 2) Characterization of cytokines present in the vaginal microenvironment; 3) Evaluation of the blood count ratios as prognostic systemic inflammatory biomarkers; 4) Correlation between MCV, HPV serotypes and cytokines.

**Methods:**

This was a retrospective, observational, multicenter, cross-sectional study. CVM analysis was performed by isolation RNA and sequencing on a NGS platform. Cytokine concentrations of CVM were obtained through Multiplex platform. Statistical analysis was performed in SPSS v 26.0. An α of 0.05 was considered statistically significant.

**Results:**

Highlighting the core of the study, CVM types of CST I and CST IV were found to influence the emergence of cervical lesions. Neutrophil-to-Lymphocyte ratio was found to impact the prognosis of ASCUS. Within CVM, *Lactobacillus* prevent the growth of other CST IV species, while the latter express symbiotic relationships with each other and show affinity for specific HPV serotypes. At last, RANTES chemokine is significantly elevated in cervicovaginal infections.

**Conclusion:**

The importance of using vaginal cytokine profiles and CVM is highlighted in the hypothesis of prevention of Cervical Neoplasia development, as well as in its use as a prognostic biomarker. Taken together, these insights are one step closer to personalized medicine.

## Background

Sexually Transmissible Infections (STI) are a heterogeneous group of diseases, inherent to human condition, associated with the core function of a species survival – reproduction. One of the most common STI is Human Papillomavirus (HPV) persistent infection. HPV is responsible, on one hand by an acute infection, that can be cleared out, while on the other hand, can lead to a chronic infection, with possible progression to cervical dysplasia and subsequent cervical neoplasia (Alizon et al., 2017).

Cervical neoplasia (CN) remains the fourth more common cancer among women, right behind breast cancer. It is accountable for around 570 000 cases and 311 000 deaths per year, according to Global Cancer Observatory 2018 data. This type of cancer affects mainly middle-aged women, particularly in countries with less resources, and it represents the major source of cancer-related deaths in women living in almost all the African continent.(Arbyn et al., 2020). The main risk factors linked to CN are smoking, oral contraceptive use and multiple gestations.

HPV *per se* can be separated in low-risk (lr-HPV) and high-risk HPV (hr-HPV), based on carcinogenic capability, with lr-HPV being correlated with anogenital warts, whereas hr-HPV is linked to cervical intraepithelial neoplasia (CIN) and CN, maNLRy due to HPV-16 and HPV-18, which represents nearly 70% of all CN. (Clifford et al., 2003; Clifford et al., 2005; Braaten and Laufer, 2008; Xiong et al., 2020) Since the onset of HPV infection, and if clearance of the virus is absent, a period of time of roughly 15 years, happens until the development of CN, walking through defined stages of CIN, corresponding to a continuous oncogenic process (Moscicki et al., 2012; Kyrgiou et al., 2017).

Approximately 80% of the population will get HPV infection, nonetheless the greater part of these infections will be cleared out by the immune system, with only a trivial proportion of HPV-infected women developing CN. (Shulzhenko et al., 2014; Shanon et al., 2017) So, CN depends on other factors, such as the microenvironment, to achieve an invasive disease. Indeed, the cervicovaginal microbiome is becoming, more clearly, a key player in the interplay between HPV infection and cervical dysplasia, CIN and CN (Gao et al., 2013; Lee et al., 2013; Brotman et al., 2014; Anahtar et al., 2015; Audirac-Chalifour et al., 2016; Di Paola et al., 2017; Kyrgiou et al., 2017; Zhang et al., 2018; Ilhan et al., 2019; Curty et al., 2020; Norenhag et al., 2020; Santella and Schettino, 2022; Cascardi and Cazatto, 2022; Kyrgiou and Moscicki, 2022).

In the cervical microenvironment microbiome arises from vertical transmission, at the time of vaginal delivery and from birth until adulthood, microbiota is unstable and suffer from considerable modifications, being highly susceptible to external influences, such as the mode of delivery (vaginal vs. C-section), ethnicity, mode of lactation (breastfeeding vs. bottle-feeding), household exposures (e.g.: siblings, pets, among others), antibiotic usage, chronic diseases, inter alia. When the microbiome gets more stable, it that can only be marginally modified by diet, level of physical activity, habits (tobacco, alcohol consumption) and pathologies (Stewart et al., 2018; Senn et al., 2020; Alpuim Costa et al., 2021).

Focusing on cervicovaginal microbiota (CVM), after the advent of 16s rRNA High-throughput sequencing technologies (16s-HTS) Ravel et al. was able to identify and divide CVM into 5 groups, baptized as Community State Types (CST). (Ravel et al., 2011) This subdivision is attributable to the low diversity of CVM (in a homeostatic state) and considers the more predominant bacteria in each composition. Thus, the main bacteria in CST I is *Lactobacillus crispatus*, in CST II is *L. gasseri*, in CST III is *L. iners* and in CST V is *L. jensenii.* In CST IV, the microbiota is slightly different, with a resemblance to bacterial vaginosis, presenting high diversity, with most anaerobic species, such as *Gardenerella, Megasphera, Sneathia, Atopobium* and *Prevotella*, combined with lower levels of *Lactobacillus* (CST I, II, III and IV). This composition is consistent with a dysbiotic state (Olmsted et al., 2003; Kyrgiou et al., 2017; Ilhan et al., 2019; Curty et al., 2020; Norenhag et al., 2020).

The “healthy” CVM comprises low diversity with widely domination of *Lactobacillus* species, as stated above. The main reason for this scenario is supported by the ability of these bacteria to produce lactic acid and hydrogen peroxide, which exert a growth control on other bacterial species and viruses (Kyrgiou et al., 2017; Tachedjian et al., 2017; Curty et al., 2020; van de Wijgert and Verwijs, 2020) CVM is modulated by hormonal changeability, associated with the reproductive cycle, acquiring a more unstable composition, during menstruation (Ravel et al., 2011; Gajer et al., 2012) and contrarywise greater stability and less diversity during pregnancy (MacIntyre et al., 2015; Kyrgiou et al., 2017). Other factors (e.g.: sexual activity, lactation, use of oral contraceptives, vaginal douche, and stress) might influence the hormonal microenvironment of cervix and vagina. (Hickey et al., 2013; Amabebe and Anumba, 2018; Curty et al., 2020) Finally, the CST-IV bacteria can enhance the concentration of pro-inflammatory cytokines (IL-17, IL-23 and Il-1β), increasing CCR5+ CD4+ T cells in vaginal mucosa, which are the targets of HIV (Gosmann et al., 2017; Torcia, 2019); as well as, increments SCFA production that, in turn, can regulate histone acetylation, which holds the possibility of reactivation of latent HIV-1 proviruses (Imai et al., 2012; Curty et al., 2020).

The primary outcome of our study was to understand the interplay between HPV infection, CVM composition and CIN stages, by comparing cervicovaginal microbiotas of healthy women without HPV, HPV infected women and women in several CIN stages. Furthermore, there was the intention to characterize the vaginal cytokine microenvironment, to evaluate the usage of blood count ratios as a prognostic biomarker of systemic inflammation and, finally, to correlate between CVM, HPV genotypes and cytokines.

## Materials and methods

### Study design and participants

This project is a retrospective, observational, multicentric and cross-sectional study, performed at the Genetics Laboratory of ISAMB, Faculdade de Medicina da Universidade de Lisboa, Dr. Joaquim Chaves, clinical analysis laboratory, and Health and Technology Research Center (H&TRC) – Escola Superior de Saúde de Lisboa, from Instituto Politécnico de Lisboa. The microbiome data was already stored in Joaquim Chaves Saúde Lab.

By assuming a 10% of significancy level, a statistical power of 90% is achieved. According to Human Papillomavirus and Related Diseases Report (Bruni et al., 2021), 865 women are diagnosed with cervical neoplasia and, by assuming a standard deviation of 0,5, it is possible to extrapolate a need for a minimum sample size number of 68 patients with, at least, 17 patients per group. Therefore, we obtained the minimum quantity of patients, adding 57 more, and so, there was a total sample size of 125 patients.

The study was divided into 4 groups:

**Control Group 1:** participant non-infected with persistent HPV and without cervical lesions;
**Control Group 2:** participant infected with persistent HPV and without cervical lesions;
**Experimental Group 1:** participant infected with persistent HPV and with ASCUS;
**Experimental Group 2:** participant infected with persistent HPV and with LSIL;

This study only includes patients with the following characteristics:

Age equal or higher than 18 years old and the informed consent from each participant.

The main exclusion criterion was the presence of any known abnormal clinical or laboratory change that, as per the investigator, might interfere with the safety or efficacy assessment or any study procedure. Patient received an informed consent and a demographic questionnaire, via email, that was fulfilled with the aim to adjust the analysis to potential confounding factors.

### HPV determination

HPV was determined in the same cervicovaginal exudate samples as the microbiota analysis. Through a clinical assay called CLART HPV 2, produced by the company Genomica, based in Spain, which made it possible to determine 35 HPV genotypes, namely 6, 11, 16, 18, 26, 31, 33, 35, 40, 42, 43, 44, 45, 51, 52, 53, 54, 56, 58, 59, 61, 62, 66, 68, 70, 71, 72, 73 81, 82, 83, 84, 85, 89.

### Microbiota analysis

The cervicovaginal microbiota samples was stored in the Joaquim Chaves Saúde laboratories, namely in ThinPrep^®^ medium and kept in refrigerator at 0°C. The Thinprep medium stabilizes the cells and microbiota, that were promptly extracted after harvesting. This analysis was performed through a PCR, with specific primers for the V1-V2 regions of the 16sRNA, due to the enhanced sensitivity and specificity (López-Aladid, 2023). The kit used was specific for 16S analysis, developed by Thermofisher. Afterwards, the samples were sequenced in the Ion GeneStudio S5 System, with Ion-Torrent Software, a system from Thermofisher Scientific. For control proposes, a sample of E. coli was used, in a concentration of 1 ng/μL. The rarefaction level was already part of the Ion-Torrent specifications. The identification of CST was performed by VALENCIA program (France and Ma, 2020), using Silva (www.arb-silva.de, s.d.) and Greengenes database (www.greengenes.secondgenome.com, s.d.).

### Identification of other micro-organisms

The determination of other microorganisms was even performed on the same cervicovaginal exudate samples as the microbiota analysis and the HPV determination.

*Mycoplasmas* spp., essentially *Ureaplasma urealyticum, Ureaplasma parvum* and *Mycoplasma hominis* were evaluated by PCR with specific probes, according to the Amplisens/Florocenosis protocol for *Mycoplasma*. *Chlamydia trachomatis* and *Neisseria gonorrhoeae* were evaluated on a COBAS 4800 CT/NG platform from a company based in Switzerland. Finally, testing to identify other etiologic agents of sexually transmitted infections was performed using a CLART STIs clinical assay from Genomica of Spain.

### Cytokine analysis

In the second and third phases of the study, approximately 1 mL of cervicovaginal exudates collected from the selected participants were analyzed by MILLPILEX MAP Human Cytokine/Chemokine Magnetic Bead Panel – Immunology Multiplex Assay (Merck-Milipore, Massachusetts, USA), divided into 2 kits (HCCBP1MAG-58K-05 and HCYTA-60K-07), with the aim understand the cervicovaginal microenvironment. The examined cytokines were:

**Pro- and anti-inflammatory cytokines (**IP10, MIP1β, RANTES, MIF, TNF-*alpha*, IL-10 and IL-6);
**Angiogenic factors (**SCF and VEGF);
**Stem-cell and metastization factors (**EGF, FGF-2).

### Clinical data

In every of the three phases, clinical data, namely protein C-reactive (PCR), neutrophil-lymphocyte ratio (NLR), platelet-lymphocyte ratio (PLR) and lymphocyte-monocyte ratio (LMR), was collected from the patient file and was subsequently incorporated into the analysis.

### Statistical analysis

Descriptive analysis was used for demographic characteristics and clinical conditions. Data was expressed as means and standard deviations for continuous variables and frequencies and percentages for categorical variables.

In the first phase and second phase, Shapiro-Wilk tests were performed, for continuous variables, with the aim to find out if normality was present. Also, Levene test was applied in continuous variables for checking homogeneity of variances. For categorical variables, Fisher test was implemented to evaluate if there is any statistically significant difference. The outcomes variables were compared between two groups (cases and controls) using statistical tests, such as Student t-test, if normality was present, or the alternative non-parametric Mann-Whitney U test (if the normality assumption is not satisfied). When the comparison is between more than 2 groups, ANOVA is performed, when normality is available, or Kruskal-Wallis test is applied, if normality is not fulfilled. *Post-Hoc* multiple comparisons, namely Scheffe and Bonferroni, were applied whenever ANOVA test resulted in statistically significant differences. Confounding variables were detected by Mantel-Haenszel statistics and adjusted with ANCOVA (Analysis of Covariance). Pearson and Spearman correlation coefficients and other association measures will be calculated to analyze relationships between continuous or ordinal variables, respectively. Also, linear relationship between variables will be analyzed using linear or logistic regression analysis (according to the nature of dependent variable).

For the third phase, when a comparison between two samples of the same individual was made, a t-student test for paired samples (in the assumption that normality is present) or a Wilcoxon test (in the assumption that normality was absent) was performed. Furthermore, when the different groups with the paired samples were compared, a one way ANOVA was performed, if normality was assumed, whereas a Friedman test was applied, when there was lacking normality.

All statistical analyses were performed using the Statistical Package for the Social Sciences (SPSS), version 24.0, for Windows. An alpha of 0.05 was considered statistically significant, and 95% confidence intervals were reported where appropriate.

## Results

### First sub-section: cervicovaginal microbiome analysis

#### Cohort characterization

As previously described, 125 women participated in this study and all their samples were collected during their medical appointments (without any modification of their normal medical routine) and analyzed by Dr. Joaquim Chaves Clinical Analysis Laboratory. Subsequently, according to their cytology results and HPV status, women were divided in 1) Control Group 1 (HPV –; normal cytology); Control Group 2 (HPV +; normal cytology); 3) Experimental Group 1 (ASCUS in cytology); Experimental Group 2 (LSIL in cytology). The whole demographic and clinical characteristics are available in **Table 1**.

**Table 1:** Demographic and clinical characteristics of first phase analysis.

	Controls		Experimental		P-value
	HPV -	HPV +	ASCUS	LSIL	
**N° of women**	79	13	21	12	
**Age (Mean ± SD)**	44 ± 11	44 ± 12	38 ± 10	35 ± 10	**0,027^1^**
**Type of HPV**	NA	**HPV 16:** 30,8% (4)	**HPV 16:** 28,6% (6)	**HPV 16:** 8,3% (1)	NA
		**HPV 33:** 15,4% (2)	**HPV 18:** 4,8% (1)	**HPV 31:** 8,3% (1)	
		**HPV 35:** 7,7% (1)	**HPV 11:** 9,5% (2)	**HPV 35:** 25% (3)	
		**HPV 52:** 30,8% (4)	**HPV 31:** 14,3% (3)	**HPV 39:** 8,3% (1)	
		**HPV 53:** 7,7% (1)	**HPV 35:** 19% (4)	**HPV 45:** 8,3% (1)	
		**HPV 56:** 7,7% (1)	**HPV 51:** 4,8% (1)	**HPV 51:** 25% (3)	
		**HPV 58:** 15,4% (2)	**HPV 52:** 4,8% (1)	**HPV 52:** 8,3% (1)	
		**HPV 66:** 7,7% (1)	**HPV 58:** 4,8% (1)	**HPV 53:** 8,3% (1)	
			**HPV 59:** 4,8% (1)	**HPV 56:** 25% (3)	
			**HPV 66:** 9,5% (2)	**HPV 58:** 16,7% (2)	
			**HPV 70:** 9,5% (2)	**HPV 82:** 8,3% (1)	
**Other Microorganisms (% of positive)**	12,7% (10)	23,1% (3)	23,8% (5)	41,7% (5)	0,079^2^
***Ureaplasma* spp. *(% of positive)***	13,9% (11)	15,4% (2)	23,8% (5)	41,7% (5)	0,138^2^
**Type of microbiome**	**CST I:** 62% (49)	**CST I:** 61,5% (8)	**CST I:** 42,9% (9)	**CST I:** 58,3% (7)	0,825^2^
	**CST III:** 1,3% (1)	**CST III:** 0% (0)	**CST III:** 4,8% (1)	**CST III:** 0% (0)	
	**CST IV-A:** 16,5% (13)	**CST IV-A:** 15,4% (2)	**CST IV-A:** 14,3% (3)	**CST IV-A:** 16,7% (2)	
	**CST IV-B:** 20,3% (16)	**CST IV-B:** 23,1% (3)	**CST IV-B:** 38,1% (8)	**CST IV-B:** 25% (3)	
**PCR (Mean ± SD)**	0,27 ± 0,26	0,39 ± 0,36	0,29 ± 0,52	0,25 ± 0,47	0,406^1^
**Neutrophil – Lymphocyte ratio (Mean ± SD)**	1,91 ± 1,02	2,26 ± 1,82	1,21 ± 0,45	2,20 ± 1,10	**0,037^1^**
**Platelet – Lymphocyte ratio (Mean ± SD)**	140,8 ± 55,5	119,5 ± 42,1	107,8 ± 40,2	135,2 ± 39,1	0,238^1^
**Lymphocyte - monocyte ratio (Mean ± SD)**	5,86 ± 2,35	6,06 ± 3,77	6,89 ± 1,95	6,03 ± 3,09	0,352^1^

^1^Application of Kruskal-Wallis test. ^2^Application of Chi-square test. All the bold values, in the p-value column, represent statistically significant results with p-value < 0,05.

NA, not applicable.

Healthy HPV negative women (n= 79) had a mean ± SD age of 44 ± 11 years, being 12,7% infected with other microorganisms, while 13,9% were infected with *Ureaplasma* spp. Regarding the type of CVM, the majority (62%) had a CST I, whereas 1,3%, 16,5% and 20,3% reported CST III, CST IV-A and CST IV-B, respectively. Protein C-reactive mean values were 0,27 ± 0,26 mg/dL. Blood count ratios, namely neutrophil-lymphocyte, platelet-lymphocyte and lymphocyte-monocyte mean values were 1,91 ± 1,02, 140,8 ± 55,5 and 5,86 ± 2,35, respectively.

The healthy HPV positive women (n= 13) had a mean ± SD age of 44 ± 12 years. A greater part was infected, mainly, with HPV 16 and/or HPV 52, with 23,1% infected with other microorganisms, while 15,4% were infected with *Ureaplasma* spp. Regarding the type of CVM, the majority (61,5%) had a CST I, whereas 15,4% and 23,1% reported CST IV-A and CST IV-B, respectively. Protein C-reactive mean values were 0,39 ± 0,36 mg/dL. Blood count ratios, namely neutrophil-lymphocyte, platelet-lymphocyte and lymphocyte-monocyte mean values were 2,26 ± 1,82, 119,5 ± 42,1 and 6,06 ± 3,77, respectively.

Women with ASCUS (n= 21) had a mean ± SD age of 38 ± 10 years. A greater part was infected, mainly, with HPV 16 and/or HPV 35, and other part (23,8%) were infected with other microorganisms, as well as with *Ureaplasma* spp. Regarding the type of CVM, the majority (42,9%) had a CST I, whereas 4,8%, 14,3% and 38,1% reported CST III, CST IV-A and CST IV-B, respectively. Protein C-reactive mean values were 0,29 ± 0,52 mg/dL. Blood count ratios, namely neutrophil-lymphocyte, platelet-lymphocyte and lymphocyte-monocyte mean values were 1,21 ± 0,45, 107,8 ± 40,2 and 6,89 ± 1,95, respectively.

Women with LSIL (n= 12) had a mean ± SD age of 35 ± 10 years. A greater part was infected, mainly, with HPV 35 and/or HPV 51 and/or HPV 56, but 41,7% were infected with other microorganisms, as well as with *Ureaplasma* spp. Regarding the type of CVM, the majority (58,3,9%) had a CST I, whereas 16,7% and 25% reported CST IV-A and CST IV-B, respectively. Protein C-reactive mean values were 0,25 ± 0,47 mg/dL. Blood count ratios, namely neutrophil-lymphocyte, platelet-lymphocyte and lymphocyte-monocyte mean values were 2,20 ± 1,10, 135,2 ± 39,1 and 6,03 ± 3,09, respectively.

#### Comparison of demographic and clinical variables between healthy women (with/without HPV) vs. ASCUS vs. LSIL

As observed in **Table 1**, in terms of demographic features, such as co-infection with other microorganisms and the type of CVM, there is no statistically significant difference between healthy women, ASCUS and LSIL, as well as, regarding clinical information, PCR, platelet- lymphocyte and lymphocyte-monocyte ratios.

Nevertheless, age appears to be a factor that is significantly dissimilar, since younger women had more infections (0,027), and the age differed significantly between healthy HPV negative women and ASCUS patients (**Table 1**).

Interestingly, neutrophils-lymphocyte ratio seems to have a statistically significant difference (p-value = 0,037) between healthy, ASCUS and LSIL women, maNLRy amid ASCUS and healthy HPV negative women, with a tendency to be increased, as well as among ASCUS and LSIL, with a tendency to be reduced (**Table 1**).

Regarding clinical characteristics, essentially PCR (p-value = 0,394), NLR (p-value = 0,127), PLR (p-value = 0,361) and LMR (p-value = 0,262), none of them modified whether HPV was present or absent.

Since the two main variables that conditionate group formation are HPV status and cytology results, there was the intention to explore if age can influence either one of the variables. From what was appraised, age influences both the susceptibility to acquire HPV (p-value = 0,01) and the cytology result itself (p-value = 0,08). Moreover, the main difference is between LSIL and normal cytology, even behind Bonferroni correction. At last, co-infection with other microorganisms (p-value = 0,650), especially with *Ureaplasma* spp. (p-value = 0,165), does not seem to influence cytology results in our population.

Furthermore, we wanted to understand if co-infection with other bacteria could affect clinical features values. Apparently, co-infection does not have an impact in PCR values (p-value = 0,184), NLR (p-value = 0,732), PLR (p-value = 0,732) and LMR (p-value = 0,585). Neither co-infection specifically with *Ureaplasma* spp. can affect PCR (p-value = 0,424), NLR (p-value = 0,777), PLR (p-value = 0,888) or LMR (p-value = 0,840).

#### Comparison of cervico vaginal microbiota between healthy (with/without HPV) vs. ASCUS vs. LSIL women

Afterwards, we intend to verify if all the variables described above might have an impact in the cervicovaginal microbiota.

First, as it is exposed in **Table 2**, *L. jensenii* (p-value = 0,022) and *Megaspherae* type 1 (p-value = 0,022) are significantly dissimilar between all groups, specially between healthy HPV negative women and LSIL, (p-value = 0,015).

**Table 2:** Cervicovaginal microbiota of first phase analysis.

	Controls		Experimental		P-value
	HPV -	HPV+	ASCUS	LSIL	
***N° of women***	79	13	21	12	
***L. crispatus***	82624,6 ± 164012,5	29187,1 ± 30320,5	59252,4 ± 118276,9	100441,7 ± 160143,6	0,568^1^
***L. gasseri***	5196,2 ± 10434,3	2630,8 ± 4673,2	2357,1 ± 6358,3	5783,3 ± 8252,5	0,506^1^
***L. iners***	2873,4 ± 18177,4	0	5842,9 ± 21454,8	1191,7 ± 2842	0,771^1^
***L. jensenii***	0	0	0	36666,7 ± 127017,1	**0,022^1^**
***A. vaginae***	68125,3 ± 173398,4	112692,3 ± 284989,9	117285,7 ± 210289,6	119500 ± 253359,8	0,641^1^
***G. vaginalis***	195148,1 ± 481161,1	409769,2 ± 857643,5	191666,7 ± 461364,8	113166,7 ± 345264,5	0,488^1^
***BVAB2***	7386,1 ± 30585,4	384,6 ± 1386,8	10880,9 ± 33123,2	28166,7 ± 65950,2	0,194^1^
***Megaspharae 1***	0	0	0	18083,3 ± 62642,5	**0,022^1^**
***Mobiluncus***	0	0	0	0	-

^1^Application of Kruskal-Wallis test. All the bold values, in the p-value column, represent statistically significant results with p-value < 0,05.

Furthermore, it is possible to comprehend that the abovementioned bacteria, essentially *L. jensenii* and *Megaspharae* type 1 are the ones that influence cytology result (p-value = 0,008), although there is an absence of impact in HPV status (p-value = 0,323), co-infection with other microorganisms (p-value = 0,328), neither with *Ureaplasma* spp. (p-value = 328).

#### Correlation between CVM and clinical and demographic data from healthy women (with/without HPV) vs. ASCUS vs. LSIL

Furthermore, it was assessed whether the CVM correlates in any way with the remaining clinical and demographic data.

Regarding clinical data, an intense and statistically significant positive correlation was observed between HPV status and cytology result (r = 0.631; P-value < 0.001), as expected. In addition, it was similarly found that the bacteria that constitute CVM had direct correlation with cytology results, namely *L. iners* (r = 0.211; P-value = 0.018), *L. jensenii* (r = 0.182; P-value = 0.042) and BVAB2 *Megasphaera* type 1 (r = 0.186; P-value = 0.038). Regarding HPV status, only the latter bacterial species were positively correlated (r = 0.183; P-value = 0.041). The possible interactions between the available laboratory values such as CRP, NLR, PLR and LMR were also evaluated. Therefore, there was an absence of relationships between the CVM and the blood count ratios, but the CRP was directly correlated with the concentration of the *G. vaginalis* bacterium (r = 0.345; p-value = 0.015). Additionally, a direct correlation was observed between the variable corresponding to the presence of other microorganisms and the concentration of *L. jensenii* (r = 0.182; P-value = 0.042).

#### Correlation between bacteria belonging to CVM

Moreover, with the aim to understand the ecology in the cervicovaginal area, we observed that *L. crispatus* showed negative, intense and statistically significant correlations, with bacteria compatible with bacterial vaginosis, namely *A. vaginae* (r = - 0.342; P-value < 0.001), *G. vaginalis* (r = - 0.465; p-Value < 0.001) and BVAB2 *Megasphaera* type 1 (r = -0.337; p-Value < 0.001), while simultaneously showing a correlation with marked positivity with *L. gasseri* (r = 0.403; p-Value < 0.001). In turn, *L. gasseri* maintained the same intense negative statistically significant correlations with *A. vaginae* (r = -0.283; p-Value < 0.001), *G. vaginalis* (r= -0.312; p-Value < 0.001) and BVAB2 *Megasphaera* type 1 (r = -0.229; p-Value = 0.01).

Finally, it was realized that species compatible with bacterial vaginosis showed positive correlations among themselves, namely between BVAB2 *Megasphaera* type 1 and *A. vaginae* (Coefficient = 0.277; P-value = 0.02), but not only that, also BVAB2 *Megasphaera* type 1 also maintained a rather vehement positive interaction with *L. jensenii* (r = 0.437; p-Value < 0.001).

#### Affinity between CVM bacteria and HPV genotype

Furthermore, the affinity between CVM and HPV genotype was evaluated. Something to highlight is the fact that *L. crispatus*, the dominant species of CST I, does not show any kind of affinity with HPV.

On the other hand, *L. gasseri* presented only a positive correlation with HPV-51 (r = 0.299; p-Value = 0.001) and *L. jensenii* correlates directly with HPV-56 (r = 0.494; p-Value < 0.001). *L. iners*, on the other hand, is positively correlated¸ and with some vehemence, with HPV-18 (r = 0.340; p-Value < 0.001), HPV-53 (r = 0.221; p-Value = 0.013) and HPV-59 (r = 0.410; p-Value < 0.001).

The species belonging to CST IV, also showed high affinity to HPV, namely *A. vaginae* with HPV-11 (r = 0.272; p-Value = 0.002) and HPV-70 (r = 0.272; p-Value = 0.002), *G. vaginalis* with HPV-16 (r = 0.186; p-Value = 0.038) and BVAB2 *Megasphaera* type 1 with HPV-16 (r = 0.213; p-Value = 0.017), HPV-18 (r = 0.268; p-Value = 0.002) and HPV-39 (r = 0.277; p-Value = 0.002).

Finally, we found that other invasive microorganisms, such as *Trichomonas vaginalis*, presented affinities with a greater number of genotypes, namely HPV-6 (r = 0.182; p-Value = 0.042), HPV-11 (r = 0.259; p-Value = 0.004), HPV-39 (r = 0.182; p-Value = 0.042), HPV-45 (r = 0.182; p-Value = 0.042), HPV-56 (r = 0.255; p-Value = 0.004), HPV-59 (r = 0.259; p-Value = 0.012), HPV-70 (r = 0.859; p-Value = 0.004) and HPV-82 (r = 0.182; p-Value = 0.042), while the presence of *Ureaplasma* spp. correlates positively with HPV-11 (r = 0.241; p-Value = 0.007), HPV-56 (r = 0.234; p-Value = 0.009), HPV-59 (r = 0.222; p-Value = 0.032) and HPV-70 (r = 0.241; p-Value = 0.007).

### Second sub-section: analysis of the correlation between MCV and the cytokine microenvironment

#### Comparison of cytokines values between healthy women (with/without HPV) vs. ASCUS vs. LSIL

Regarding cytokines, the mean values per group are described in **Table 3**.

**Table 3:** Cytokines analysis of second phase.

Cytokines (pg/mL)	Controls		Experimental Groups		P-values
	HPV – (16)	HPV+ (7)	ASCUS HPV + (6)	LSIL HPV + (3)	
***EGF***	12,96 ± 19,41	30,41 ± 38,44	9,18 ± 15,14	18,72 ± 27,23	0,695^1^
***IL-10***	15,12 ± 4,13	20,61 ± 18,41	13,10 ± 2,22	17,46 ± 2,53	0,499^1^
***MIP-1β***	0,38	30,35 ± 79,30	0,38	0,38	0,446^1^
***RANTES***	7,69 ± 4,49	16,30 ± 24,83	6,26 ± 0,76	6,92 ± 0,76	0,890^1^
***IP-10***	9,53 ± 12,96	23,40 ± 26,91	2,6	33,43 ± 31,74	0,156^1^
***FGF-2***	167,25 ± 94,48	245,34 ± 253,08	67,01 ± 94,68	198,32 ± 50,11	0,147^1^
***IFG-γ***	11,43 ± 13,41	14,44 ± 15,56	6,15 ± 9,34	17,54 ± 4,4	0,177^1^
***MIF***	77,88 ± 201,93	27,4	27,4	27,4	0,900^1^
***IL-6***	3,23 ± 3,53	13,5 ± 15,38	3,54 ± 3,36	1,91 ± 2,21	0,147^1^
***TNF-α***	1,97 ± 2,55	1,3	1,3	1,3	0,701^1^
***SCF***	6,9	17,5 ± 28,04	6,9	6,9	0,446^1^
***VEGF***	49,92 ± 144,88	13,7	13,7	13,7	0,900^1^

^1^Kruskal-Wallis test. All the values in bold are statistically significant results with a p-value < 0,05.

As it can be seen in **Table 3**, there are no statistically significant changes in any of the cytokines evaluated.

It was then assessed whether several variables, namely age, HPV status, cytology result, presence of other microorganisms, presence of *Ureaplasma* spp. and type of MCV, significantly influenced cytokine concentrations. It was concluded that most variables did not significantly affect cytokine concentrations (p-Value > 005), apart from the presence of microorganisms which apparently specifically affects the RANTES chemokine (p-Value = 0.033).

#### Correlation between CVM and cytokines in healthy women (with or without HPV) vs. ASCUS vs. LSIL

Furthermore, aimed to understand the relationship between the constituent bacteria of the various types of MCV.

Although most bacteria and cytokines did not show statistically significant correlations, it is possible to highlight the existing negative correlation between the bacteria *L. iners* and FGF-2 (r= - 0.366; p-Value = 0.043), as well as the intense positive correlation between *L. gasseri* and SCF (r = 0.558; p-Value = 0.001). Additionally, one of the main bacteria belonging to the CST IV-A microbiotic composition, *Atopobium vaginae* correlates negatively with IP-10 (r = - 0.365; p-Value = 0.044) and positively with IL-6 (r = 0.388; p-Value = 0.028).

Additionally, correlations were assessed between cytokines and other clinical data, namely cytology result, HPV status, presence of microorganisms, presence of *Ureaplasma* spp. and type of CVM. Therefore, there were no statistically significant correlations between most variables, except for the variable related to the presence of microorganisms, with the chemokine RANTES (r = 0.401; p-Value = 0.028) and the type of CVM, which correlated directly with the cytokine IL-6 (r = 0.429; p-Value = 0.014).

## Discussion

Despite the unequivocal development of the last few years, regarding prognosis, prevention, and treatment of cervical neoplasia, it is still a pathology with a considerable prevalence and associated morbidity and mortality, especially in low-income countries. One of the major recent objectives of the Medical Community is the eradication of this disease, with an underlying symbolism associated with the cure for cancer.

Thus, this study aimed to identify new prognostic biomarkers, to assist the gynecologist physician in his/her clinical decision-making process while simultaneously aiming to understand the role of MCV both in the pathophysiology, as well as in its use for prevention and/or therapy in cervical neoplasia.

According to the results obtained, the most prevalent type of CVM in each of the groups is CST I, dominated by *L. crispatus*, with the following prevalences: 62%, 61.5%, 42.9% and 58.3%, corresponding to healthy women without and with HPV, women with ASCUS and women with LSIL, respectively. This type of CVM is more present in healthy individuals and belonging to European populations (Curty et al., 2020). Comparing with other geographical regions, we found different prevalences, namely a predominance of CST type III in Asia, while in Africa and Australia the dominance is clear for CST type IV (Ravel et al., 2011; Chico et al., 2012; Bradshaw et al., 2013; Kyrgiou et al., 2017; Curty et al., 2020; Onywera et al., 2021).

Furthermore, the impact that the age of patients in the various study groups might have on the interpretation of the results was assessed. We observed that healthy HPV-negative and/or HPV-positive women had a mean age of 44 years, while the remaining women had 38 and 35 years, respectively. When this dissimilarity was further investigated, it was noticed that it was mainly between HPV-negative healthy women and women with ASCUS. Moreover, it was found that this inequality, in terms of age, was significant not only in relation to the cytology result, but also in the susceptibility to acquire HPV. In this case, the interaction is more marked between healthy women and women with LSIL. This relationship agrees with another study, which reports a 50% and 75% risk of acquiring HPV at the mean ages of 20 and 30 years, respectively (Burger et al., 2017). Additionally, another author realized that, as women age, HPV-induced cervical modifications decrease, reaching only 38% above 46 years of age (Kainz et al., 1995), which coincides with the results that we obtained. In addition, it should be noted that many older women have not received the HPV vaccine, contrary to younger women, which may influence both the susceptibility to HPV and the cytology result itself because, if the woman is infected with HPV, it will probably be a genotype with less carcinogenic risk.

Blood count ratios were analyzed with the aim of identifying possible prognostic biomarkers. According to the literature, we can understand that the ratio showed greater prominence and more reliable results is the neutrophil-lymphocyte ratio. Indeed, several studies show that a higher NLR correlates with a worse prognosis, namely through a decrease in both overall survival and disease-free survival (Templeton et al., 2014; Lima et al., 2021). In addition, an increased risk of locoregional metastasis, lymphovascular invasion and tumor size has even been assessed (Wu et al., 2017; Ma et al., 2018). Moreover, it was found that even applying this ratio in CIN, namely regarding its post-excision recurrence, an elevation of this marker, would be related to a decrease in the disease-free interval, which means, an increment in the risk of recurrence (Farzaneh et al., 2019). Given that our study was based on early stages of CIN, it is not possible to substantiate the existing evidence. However, there as a summing of further evidence to this prognostic biomarker, particularly regarding the clinical uncertainty associated with an ASCUS cytology. In this study, it was possible to understand that the NLR’s differed significantly between the various groups. When this dissimilarity was further investigated, the conclusion reached was that it essentially resided among women in the ASCUS group, healthy women with negative HPV and women with LSIL. Thus, we understand that an increase in the NLR in a woman with ASCUS relates to a progression to LSIL, while a decrease in the NLR, in the same woman, is associated with a regression to a normal cytology. It therefore helps the gynecologist to decide what the next step should be when faced with a diagnosis of ASCUS.

Furthermore, the associations between the constituent bacteria of CVM and the numerous available parameters were scrutinized. In this way, it was assessed the interaction between CVM and the cytology result itself.

It was possible to observe that both *L. jensenii* and *Megasphaera* type 1 were significantly different between the various study groups, essentially between HPV negative healthy women and women with LSIL. The same positive correlation was found between the above bacteria and the cytology result. These results are in line with the existing literature. One of the studies, which associated CVM with cytology result, showed that a CVM of CST type IV, where *Megasphaera* type 1 is included, increased the risk of developing LSIL by 2-fold, HSIL by 3-fold and CN by 4-fold, respectively (Mitra et al., 2015; Mitra et al., 2016; Kyrgiou et al., 2017). Other studies report similar results, where an CVM is intrinsically associated with a higher risk of developing more advanced CIN and, consequently, CN (Oh et al., 2015; Piyathilake et al., 2016; Łaniewski et al., 2018; Łaniewski et al., 2020; Curty et al., 2020; Norenhag et al., 2020). As for the issue of *L. iners*, the dominant species of CST III, despite being a *Lactobacillus* spp., it is classified as a transitional species for microbiotic dysbiosis, given that in some studies, it presents a risk of interaction with other species integrating CST IV and, therefore, increases the risk of developing LSIL and HSIL (Kinney et al., 1998; Kyrgiou et al., 2017). This can be explained by the smaller genome of *L. iners*, which leads to the production of L-lactic acid, in contrast to the other members of *Lactobacillus* spp. which produce mainly D-lactic acid. The underlying problem is the different biological actions inherent to L-lactic acid and D-lactic acid, with D-lactic acid having anti-inflammatory characteristics, as opposed to L-lactic acid, which has pro-inflammatory characteristics, with the capacity to reactivate latent viral infections. Complementarily, *L. iners* also presents the ability to produce inerolysin, similar to vaginolysin, both toxins that allow the formation of pores in the vaginal membrane, with subsequent facilitation of HPV infection and, ultimately, cytological alteration(Rampersaud et al., 2011; Kyrgiou et al., 2017; Petrova et al., 2017; Ragaliauskas et al., 2019; Curty et al., 2020; Pleckaityte, 2020).

As for *L. jensenii*, it is positively correlated with advanced stages of cervical lesions, as well as increased susceptibility of infection by other microorganisms. Spurbeck et al. demonstrated the anti-inflammatory properties of *L. jensenii* regarding the inhibition of *Neisseria gonorrheae* infection (Spurbeck and Arvidson, 2010). Another study showed that the presence of *L. jensenii* would allow achieving cure, in patients with bacterial vaginosis, about 1.67 times compared to the control (Mitchell et al., 2012). Therefore, our results are dissonant with the literature. Thus, our database was explored with the purpose to find an explanation, which justifies this dissimilarity. The most plausible explanation is that only 1 of 125 women participating in the study had a CVM with identification of *L. jensenii*. Therefore, the statistical power is too low to draw conclusions.

Furthermore, a direct correlation was found between the bacteria *L. crispatus* and *L. gasseri*, which was already expected, since although *L. crispatus* is the dominant species in most microbiotas. It is possible to observe a symbiotic relationship with other *Lactobacillus* species in order to maintain the homeostasis of the cervicovaginal microenvironment. Furthermore, both bacteria possess the ability to inhibit cell proliferation and induce apoptosis, essentially in tumor cells, making them crucial for protection against both infectious and oncological pathologies (Motevaseli et al., 2013). However, *L. crispatus* show negative correlations with species belonging to CST IV, compatible with microbiotic dysbiosis, namely *A. vaginae*, *G. vaginalis* and BVAB2 *Megasphaera* type 1. These associations are plausible given the very functioning of *L. crispatus* which possesses several strategies to reduce colonization by other more deleterious species. Firstly, it has the capacity to decrease the pH of the medium, converting itself into a medium with low pH, through the production of D-lactic acid, by a mechanism whose substrate is glycogen, deposited in the vaginal walls, resulting from the action of oestrogen. It can then also produce hydrogen peroxide and bacteriocins. Taken together, they prevent the growth of other harmful bacterial species (Kyrgiou et al., 2017; Tachedjian et al., 2017; Curty et al., 2020; van de Wijgert and Verwijs, 2020). Therefore, our results are in agreement with the literature. Furthermore, *L. gasseri* also expresses the same negative correlations with *A. vaginae*, *G. vaginalis* and BVAB2 Megasphaera type 1, but with less intensity than *L. crispatus*. In turn, something not yet described in the literature, was the positive interaction between BVAB2 *Megasphaera* type 1 with *A. vaginae*. Apparently, the deleterious species present a symbiosis among themselves and, when there is a significant depletion of *Lactobacillus* spp. and allow their emergence and growth, these species assist the translocation of other species, with similar characteristics, thus increasing their dominance in the medium and, leading to the conversion to a CST type IV microbiota. Looking at other data, it may be possible to explain the resistance to probiotic colonization in the cervicovaginal region or the resistance provided to the treatment of bacterial vaginosis (Mitchell et al., 2012; Srinivan et al., 2015; Kyrgiou et al., 2017; Łaniewski et al., 2020; Curty et al., 2020).

Then, it was checked, in a more detailed way, if CVM had affinity for the various genotypes of HPV causing infection. Thus, it was shown that *L. crispatus* does not show any affinity for any HPV genotype, thus constituting the most resistant type of CST against HPV infection. However, a CVM dominated by *L. gasseri* had a higher risk of being infected by an HPV of genotype 51, whereas a CST type V MCV, dominated by *L. jensenii*, had a higher risk of being infected by HPV-56. In turn, *L. iners*, being a transitional species, shows affinity for a higher number of genotypes, namely HPV-18, HPV-53 and HPV-59. Therefore, it was possible to conclude that all *Lactobacillus* spp. that show some kind of affinity, only correlate with the most common genotypes in normal cytology and low-grade lesions (So et al., 2019). However, it was observed that the remaining species present in CST IV, the presence of infection by other microorganisms and even colonization by *Ureaplasma* spp. manifested a greater number of affinities with various HPV genotypes. However, they were correlated to genotypes more present in HSIL, increasing the risk of developing CN (So et al., 2019). Of note, this interaction between CVM and affinity for certain HPV genotypes had not been described in the literature.

Finally, the focus was on a topic little explored in the literature, namely the characterization of the cervicovaginal microenvironment regarding the cytokines present. Therefore, when analyzing the literature on the topic, we found that one study reported that a state of dysbiosis associated essentially with CST IV expressed an increase in pro-inflammatory cytokines, especially TNF-α, IL-6, IL-8, RANTES, IFG-γ and GMC-SF, while simultaneously manifesting a decrease in the concentration of cytokines with anti-inflammatory characteristics, such as IP-10, compared to women with so-called “healthy” CST (Hedges et al., 2006; Novak et al., 2007; Anderson et al., 2011; Anahtar et al., 2015; Kyrgiou et al., 2017; Łaniewski et al., 2018). Another cross-sectional study evaluated women with CN or dysplasia and healthy women with or without HPV infection and assessed the cervicovaginal microenvironment that had multiple pro-inflammatory cytokines (IL-36γ, IP-10, MIP-1β and RANTES), haematopoietic (FLT3 ligand), growth factors and angiogenic factors (FGF-2, SCF, TRAIL), hormones (prolactin), chemokines (MIF and TNF-α) and osteopontin. All this microenvironment was found to correlate positively with vaginal inflammation, but also with microbiotic dysbiosis (Łaniewski et al., 2019; Łaniewski et al., 2020). Unfortunately, given the tiny sample size where cytokines were assessed (n= 39), it was not possible to obtain the vast majority of the aforementioned correlations. However, we were able to perceive the interaction between infection by other microorganisms and the chemokine RANTES. This chemokine, also called CCL5, is produced by platelets, and has been shown to play an important role in cancer progression, namely in invasion, metastasis, angiogenesis, and immune cell infiltration. It appears to function as a crucial regulator, allowing two-way communication between the microenvironment, modulated by the microbiota, and tumor cells. Furthermore, it promotes macrophage recruitment, collagen deposition and tumor relapse (Walens et al., 2019). Consequently, it is present from the early stages of the tumor and therefore could be an important chemokine for understanding tumor development.

## Conclusion

The main limitations are the small number of confounding factors that may influence the results, as well as the small number of valid samples for cytokine analysis, which greatly reduced the information that we could have obtained. Furthermore, all the women only performed cytology, since the majority of cervical lesions were in the early stages, without indication for cervical biopsy, therefore it is a limitation itself, since there is not any histological confirmation of the abovementioned lesions. At last, since this is a retrospective project, there was an absence of information regarding the clinical status of the woman (such as immunosuppression), limiting the extrapolation of knowledge, since it can influence cervicovaginal microbiota composition and cervical neoplasia development.

In short, this study might contribute for another step in the development of a more personalized medicine, with more effective and efficient results and with better care for patients. Additionally, it has brought us closer to one of the most pressing challenges, namely the eradication of cervical cancer.

## Data availability statement

The raw data supporting the conclusions of this article is not available in order to protect patient confidentiality. Therefore, only the database containing the bacterial concentrations in each sample can be shared. This data can be accessed in the **Supplementary Material**.

## Ethic statement

This study was performed in accordance with the Declaration of Helsinki and was approved by Centro Hospitalar Universitário Lisboa Norte (CHULN) and Centro Académico de Medicina de Lisboa (CAML) ethics committee.

## Author contributions

JGG-N, AM, MBi, and MCB conceived the study and oversaw the sample acquisition and maintenance. JGG-N, AM, MC, MP, AS, HP, CC, and MCB contributed to data collection and analysis. JGG-N and AM participated in manuscript writing. JGG-N, AM, and MCB oversaw clinical sample and MCB the data collection. LV, CG, and MBr contributed to the cytokine analysis and manuscript reading. All authors contributed to the article and approved the submitted version.
